# Aligned Collagen-CNT Nanofibrils and the Modulation Effect on Ovarian Cancer Cells

**DOI:** 10.3390/jcs5060148

**Published:** 2021-06-02

**Authors:** Wen Li, Naiwei Chi, Elwin D. Clutter, Bofan Zhu, Rong R. Wang

**Affiliations:** Department of Chemistry, Illinois Institute of Technology, 3101 S. Dearborn St., Chicago, IL 60616, USA;

**Keywords:** collagen-CNT, aligned nanofibrils, cell–matrix interaction, SKOV3, EMT

## Abstract

Fibrillar collagen is a one-dimensional biopolymer and is the most abundant structural protein in the extracellular matrix (ECM) of connective tissues. Due to the unique properties of carbon nanotubes (CNTs), considerable attention has been given to the application of CNTs in developing biocomposite materials for tissue engineering and drug delivery. When introduced to tissues, CNTs inevitably interact and integrate with collagen and impose a discernible effect on cells in the vicinity. The positive effect of the collagen-CNT (COL-CNT) matrix in tissue regeneration and the cytotoxicity of free CNTs have been investigated extensively. In this study, we aimed to examine the effect of COL-CNT on mediating the interaction between the matrix and SKOV3 ovarian cancer cells. We generated unidirectionally aligned collagen and COL-CNT nanofibrils, mimicking the structure and dimension of collagen fibrils in native tissues. AFM analysis revealed that the one-dimensional structure, high stiffness, and low adhesion of COL-CNT greatly facilitated the polarization of SKOV3 cells by regulating the β−1 integrin-mediated cell–matrix interaction, cytoskeleton rearrangement, and cell migration. Protein and gene level analyses implied that both collagen and COL-CNT matrices induced the epithelial–mesenchymal transition (EMT), and the COL-CNT matrix prompted a higher level of cell transformation. However, the induced cells expressed CD44 at a reduced level and MMP2 at an increased level, and they were responsive to the chemotherapy drug gemcitabine. The results suggested that the COL-CNT matrix induced the transdifferentiation of the epithelial cancer cells to mature, less aggressive, and less potent cells, which are inapt for tumor metastasis and chemoresistance. Thus, the presence of CNT in a collagen matrix is unlikely to cause an adverse effect on cancer patients if a controlled dose of CNT is used for drug delivery or tissue regeneration.

## 1. Introduction

Carbon nanotubes (CNTs) are unique nanomaterials whose integration with biological materials have inspired the development of CNT-based composites for applications in tissue engineering, organ regeneration, biosensors, and drug delivery for cancer treatments [1–6]. In our previous studies, we incorporated functionalized CNT in silk protein to generate biocomposite fibers by electrospinning. The addition of a minute amount of CNT not only reinforced the strength of the scaffolds, but also rendered the fibers electrically conductive, which was utilized to stimulate fibroblasts for improved collagen synthesis for tissue repair [7,8]. We also generated the collagen-CNT (COL-CNT) gel and fibril matrices, which were found to be biocompatible and induce the accelerated neural differentiation of both embryonic stem cells and adult stem cells [9–11]. Others also reported that CNT and CNT-based polymer matrices can serve as effective drug carriers, facilitate the regeneration of neural networks, and promote bone differentiation and bone regeneration mineralization [3,4,6]. The health risk of CNTs has been a concern. Particularly, when they are released from their integrated scaffolds and implants, CNTs may disperse and redeposit in the physiological environments and induce local inflammatory reactions, causing cell apoptosis [3,4]. CNT accumulation at the tumor sites has been reported when CNT-conjugated RDG peptides and water-soluble polymers were employed as drug delivery carriers [1,12].

In the stroma of tumor tissues, collagen I is the most abundant component of an extracellular matrix (ECM). A characteristic feature of fibrillar collagen I in a stromal tissue, and generally in the extracellular matrix (ECM) of connective tissues, is that it forms densely packed, locally aligned fibrils [13–15]. These fibrils were thought to coax cell migration and potentially induce the epithelial–mesenchymal transition (EMT) in epithelial carcinoma [16,17], characterized by cells losing the epithelial features, gaining migratory properties, and shifting toward the mesenchymal phenotype associated with the regulation of a set of epithelial–mesenchymal biomarkers [18,19]. In in vitro studies, aligned polycaprolactone and polylactic acid fibers were reported to induce EMT processes in glioblastoma and breast cancer cells [13,20–22]. It was also reported that condensed collagen fibril bundles in tumor tissues markedly increased the matrix rigidity, which could promote EMT and cancer metastasis due to the presence of cancer stem cells among the transformed cells [20–23]. Owing to the high mechanical strength and one-dimensional structure, CNTs present in stromal tissues, either released from drug carriers or tissue engineering scaffolds, likely integrate with collagen to form stiffer collagen-CNT (COL-CNT) composite fibrils, which may impact EMT and tumor aggressiveness.

In this work, we aimed to reveal how COL-CNT fibrils, as the extracellular matrix, modulate the cell behavior and impinge upon the EMT process. SKOV3, a highly mechanosensitive ovarian cancer cell line, was used as a model system in the study. Aligned collagen and COL-CNT fibril matrices were generated by using a simple epitaxial growth method, in which collagen molecules were guided by the crystalline orientation of mica and self-assemble into unidirectionally aligned nanofibrils [24–27], mimicking the structure and dimension of collagen fibrils in native tissues. It was found that CNTs have the dual effect of modulating both biochemical and biophysical cues of the collagen matrix to induce extensive cell polarization, fast migration, and decreased cell adhesion. This prompts an excessive EMT process and gives rise to the transdifferentiation of the epithelial cancer cells to a mature, less aggressive phenotype, which was proven to be sensitive to the chemotherapy drug. The information derived from the study inspires the development of new methods to modulate the stroma of carcinoma for reducing the aggressiveness of tumor cells.

## 2. Materials and Methods

### 2.1. Matrix Preparation

Collagen I derived from rat tail tendon (BD Biosciences, Bedford, MA, USA) was dissolved in 0.1% acetic acid. The solution was diluted to a final collagen concentration of 35 μg/mL in 10× PBS buffer containing 1 N of NaOH to achieve pH 9 for an effective collagen fibril assembly following the manufacturer’s protocol. In addition, 400 mM of KCl was added to promote collagen alignment [24–27] on Muscovite mica disk (Grade V1, Ted Pella, Inc., Redding, CA, USA). A drop of 30 μL of collagen solution was cast on a freshly cleaved mica surface and was incubated at 37 °C overnight to achieve collagen gelation. After rinsing with PBS, the collagen fibrils on mica were subjected to AFM imaging at high resolution or served as matrices for cell culture (see below). Single-walled carbon nanotubes (SWCNTs) were purchased from HELIX Material Solution (Richardson, TX, USA). Raw SWCNTs were oxidized as reported previously [7–11]. In brief, raw SWCNT was sonicated in a solution of 8 M of H_2_SO_4_ and 8 M of HNO_3_ at 70 °C for 3 h. The SWCNT suspension was then centrifuged at 1398× *g* for 10 min, followed by collecting and filtering the supernatant with a 0.22 μm Isopore membrane (Millipore, Billerica, MA, USA). The CNT left on the membrane was then re-suspended in DI water, and the procedure was repeated until a pH of 7 was achieved. The oxidized CNT was collected as powder after drying in a vacuum oven overnight at 70 °C. The oxidized SWCNT was added to 35 μg/mL of collagen solution to achieve a final CNT concentration of 0.175 μg/mL. Chemical crosslinking of SWCNT with collagen was catalyzed by adding 0.02 M of 1-ethyl-3-(3-dimethylaminopropyl)carbodiimide hydrochloride (EDC) in 2-(N-morpholino) ethanesulfonic acid (MES) (0.2 M, pH = 6) to the collagen solution. The oxidation of CNT was confirmed by FTIR with peaks at 3400 (hydroxyl group) and 1700 cm^−1^ (carbonyl group), characteristic for carboxyl groups, which are absent in raw CNT. The conjugation of CNT to collagen was confirmed by the shift in the amide I and II bands. Aligned COL-CNT fibril matrices were prepared on a freshly cleaved mica surface following the same procedure as above.

As a control, a featureless, soft matrix formed by coating 0.1% gelatin (Fisher scientific, Fairlawn, NJ, USA) on a mica surface was used in parallel experiments to delineate the distinctive cell responses to an aligned fibril matrix and a homogenous matrix.

### 2.2. Cell Culture and Characterization

Human ovarian cancer cell line SKOV3 from ATCC (Manassas, VA, USA) was used in this study. The cells were grown in 35 mm petri dishes in Dulbecco’s minimum essential medium (DMEM) supplemented with 10% fetal bovine serum (Hyclone, Logan, UT, USA) and 1% nonessential amino acid. The medium was changed every other day, and the cells were passaged every 3 days. At confluence, cells were trypsinized and plated on target matrices with a seeding density of 5000 cells/cm^2^.

The cell polarity on various matrices was characterized by the cell length-to-width ratio, which was derived from the cell length and cell area measured from 20× optical images using ImageJ software [28]. A wound-healing assay using a culture insert (Applied Biophysics, Troy, NY, USA) was employed to study cell migration. SKOV3 cells were seeded in both chambers of a culture insert, which was placed on the selected matrices. The two chambers were separated by 500 μm. After the culture insert was removed at 5 h post-plating of the cells, sequential optical images were collected to monitor cell migration to fill the gap. The cell migration rate was evaluated in the direction of fibril alignment.

Cell viability in the absence and presence of the chemotherapy drug gemcitabine at a dose of 20 ng/mL was tested by CellTiter 96^®^ Aqueous One Solution Cell Proliferation Assay (MTS) (Promega, Madison, WI, USA). The cells grown on the matrices were subjected to the drug for three days, and they were then cultured in drug-free medium for four days before the viability tests were carried out. The percentage of metabolically active cells was determined with respect to the culture in the absence of gemcitabine.

### 2.3. Immunofluorescent Imaging

Cells grown on various matrices were fixed by 4% paraformaldehyde in PBS, permeabilized with 0.1% Triton X-100 in PBS for 10 min, and blocked by 1% BSA in PBST (PBS-0.5% tween 20). The primary antibodies used in this study included rabbit anti-β1-integrin (Santa Cruz Biotechnology, Dallas, TX, USA, 1:100 dilution), rabbit anti-E-cadherin (Abcam, MA, USA, 1:25 dilution), and mouse anti-vimentin (Abcam, MA, 1:200 dilution). Secondary antibodies conjugated with Alexa Fluor 594 and 488 fluorescent dyes were purchased from Invitrogen (Carlsbad, CA, USA) and used at 1:200 dilution. The slides were then subjected to fluorescent imaging using a Nikon TE-U 2000 microscope. For each experiment on a particular marker, imaging parameters, such as the exposure time, gain value, image size, and magnification, were kept constant through the entire study for all samples. Quantitative analyses were performed using ImageJ software (NIH free download). Background signals, measured in the absence of primary antibody, were subtracted for fluorescence intensity analysis.

### 2.4. AFM Measurements

AFM imaging was carried out using a multimode Nanoscope IIIa AFM (Veeco Metrology, Santa Barbara, CA, USA), equipped with a J-scanner. Images of the matrices were collected in 1× PBS buffer in fluid tapping mode using Si_3_N_4_ tips at a resonance frequency of 8–10 kHz. In collecting images of SKOV3 cells, the cells were gently fixed with 4% paraformaldehyde in PBS for 3 min and imaged in fluid contact mode in 1× PBS buffer.

The stiffness of the matrices and cells, characterized by the Young’s modulus (E-value), was derived from the force–distance curves collected using Si_3_N_4_ probes in fluid contact mode in 1× PBS buffer [14,15]. The spring constant of the cantilevers was 0.030 ± 0.002 N/m, calibrated using reference cantilevers with known spring constants. From each force–distance curve, the E value was derived using the Hertzian model with the AFM tip modeled as a nano-indenter to probe a one-dimensional material for fibrous matrices or a flat, infinite surface for the cells, the gelatin, and the plastic surfaces [29,30]. Histograms to illustrate the statistics of fibril or cell elasticity were generated based on E values derived from at least 120 force curves on 3 different samples.

Cell adhesion to the matrices was measured using an AFM probe pre-modified by a single cell. Cell attachment was achieved following the procedure reported by Sariisik et al. [31] with slight modification. In brief, cells grown to 80% confluence were trypsinized and centrifuged before being re-suspended in a fresh medium. A drop of the cell suspension at a seeding density of 4000 cells/cm^2^ was placed on a glass cover slip pre-coated in bovine serum albumin (BSA). An AFM probe was functionalized by poly D-lysine (PDL, Millipore, Burlington, MA, USA). Due to the stronger adhesion of a cell to PDL than to BSA, a medium-sized, round-shaped, healthy cell was transferred from the cover slip to the AFM probe. The presence of the cell on the AFM probe was confirmed by optical imaging before and after the adhesion force measurements. Force–distance curves were recorded while the piezo traveled up to 4 μm at a z-scan rate of 3 μm/s. The adhesion peak appearing in the retraction curve quantifies the strength of cell adhesion to a matrix.

### 2.5. RT-qPCR Analysis

Total RNA was extracted from SKOV3 cells using a PureLink^®^ RNA Mini Kit (Ambion, Grand Island, NY, USA) after the cells were cultured on the selected matrices for 3 days. Reverse-transcription was carried out using a SuperScript^®^ III First-Strand Synthesis System (Invitrogen, Carlsbad, CA, USA). RT-qPCR was performed using an ABI Prism 700 with TaqMan^®^ Gene Expression Master Mix and TaqMan^®^ Gene Expression Assays (Life Technology, Madison, WI, USA). Primers for the following genes were used in this study: CD44 (Hs01075861), CDH1 (Hs01023894), SNA1 (Hs00195591), VIM (Hs00958111), and MMP2 (Hs01548727). GAPDH (Hs99999905) was used as an endogenous reference. Data analysis was carried out using the 2^−ΔΔCT^ method for relative quantification based on three replicates. The expression level of a target gene in cells on a specific matrix was relative to that in cells on the petri dish before the cells were passaged and re-seeded on the target matrices.

## 3. Results

### 3.1. Aligned Collagen and COL-CNT Fibrils with Matrix-Mediated Cell Polarization and Migration

**Figure 1.**
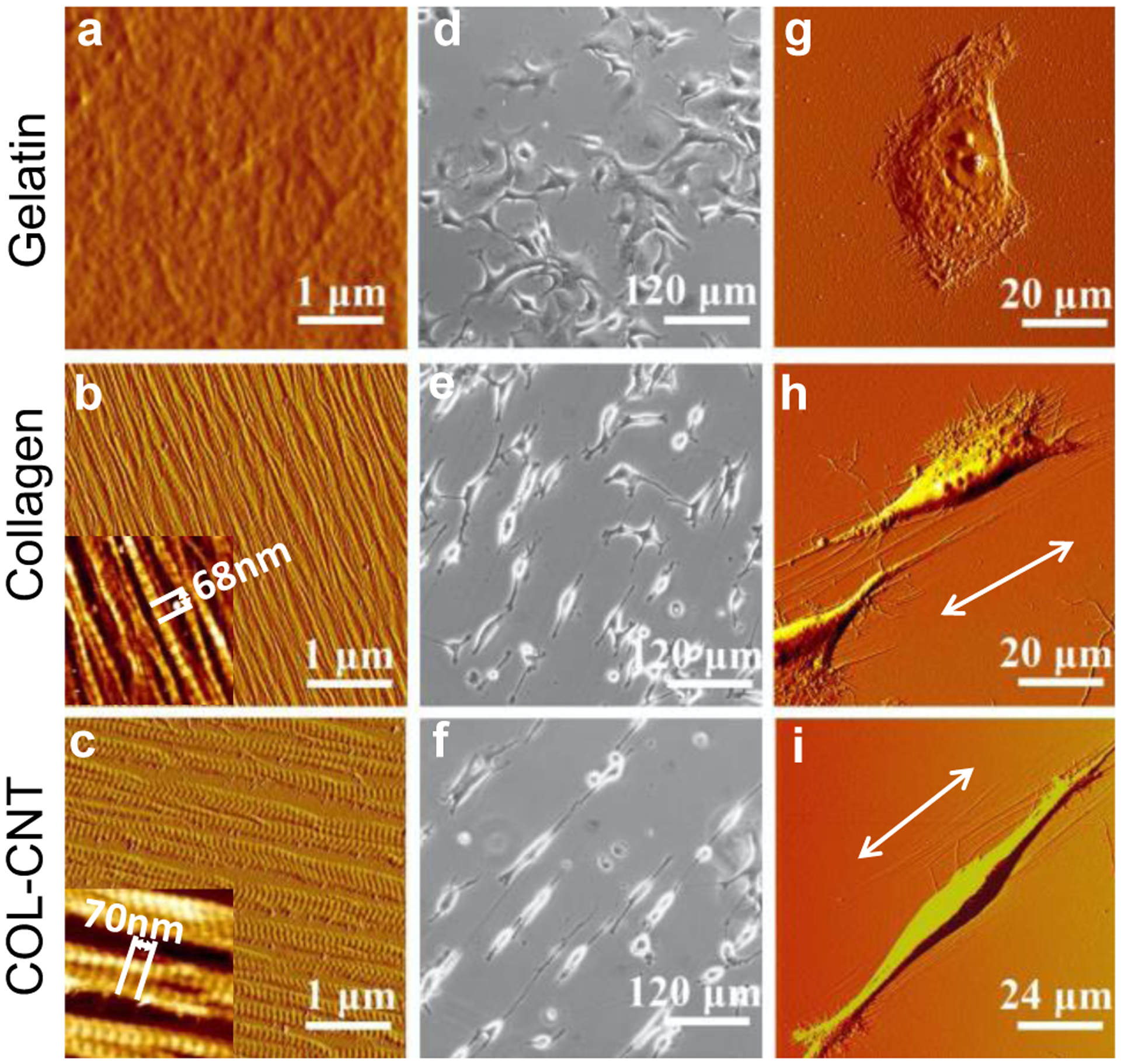
Characterization of the selected matrices and the cell growth. (**a**–**c**) AFM images (in amplitude mode) of gelatin (**a**), collagen (**b**) and COL-CNT matrices (**c**). The insets (1.6 × 1.6 μm^2^) highlight the distinctive D-period for collagen and COL-CNT fibrils. (**d**–**f**) Phase images of SKOV3 cells 24 h post-plating on the matrices. (**g**–**i**) AFM images (in amplitude mode) illustrating individual SKOV3 cells on the matrices. The arrows indicate the direction of fibril alignment.

AFM images of gelatin, collagen, and COL-CNT fibrils are shown in Figure 1a–c. Both collagen and COL-CNT assembled into unidirectionally aligned fibrils with uniform coverage on mica substrates. While collagen fibrils exhibited widths of 95 ± 22 nm, COL-CNT fibrils were much thicker (276 ± 50 nm), consistent with our structural analysis of collagen and COL-CNT fibrils in gels in the previous reports [9–11]. The characteristic collagen D-period (insets of Figure 1b,c), which signifies the proper assembly of collagen molecules into fibrils, was measured to be 68 ± 1 nm for collagen and 70 ± 1 nm for COL-CNT with a significant difference (*p* < 0.001) based on the *t*-test [11]. On the other hand, gelatin formed a smooth, featureless layer on mica with a maximum height difference of 4 nm (Figure 1a).

**Figure A1.**
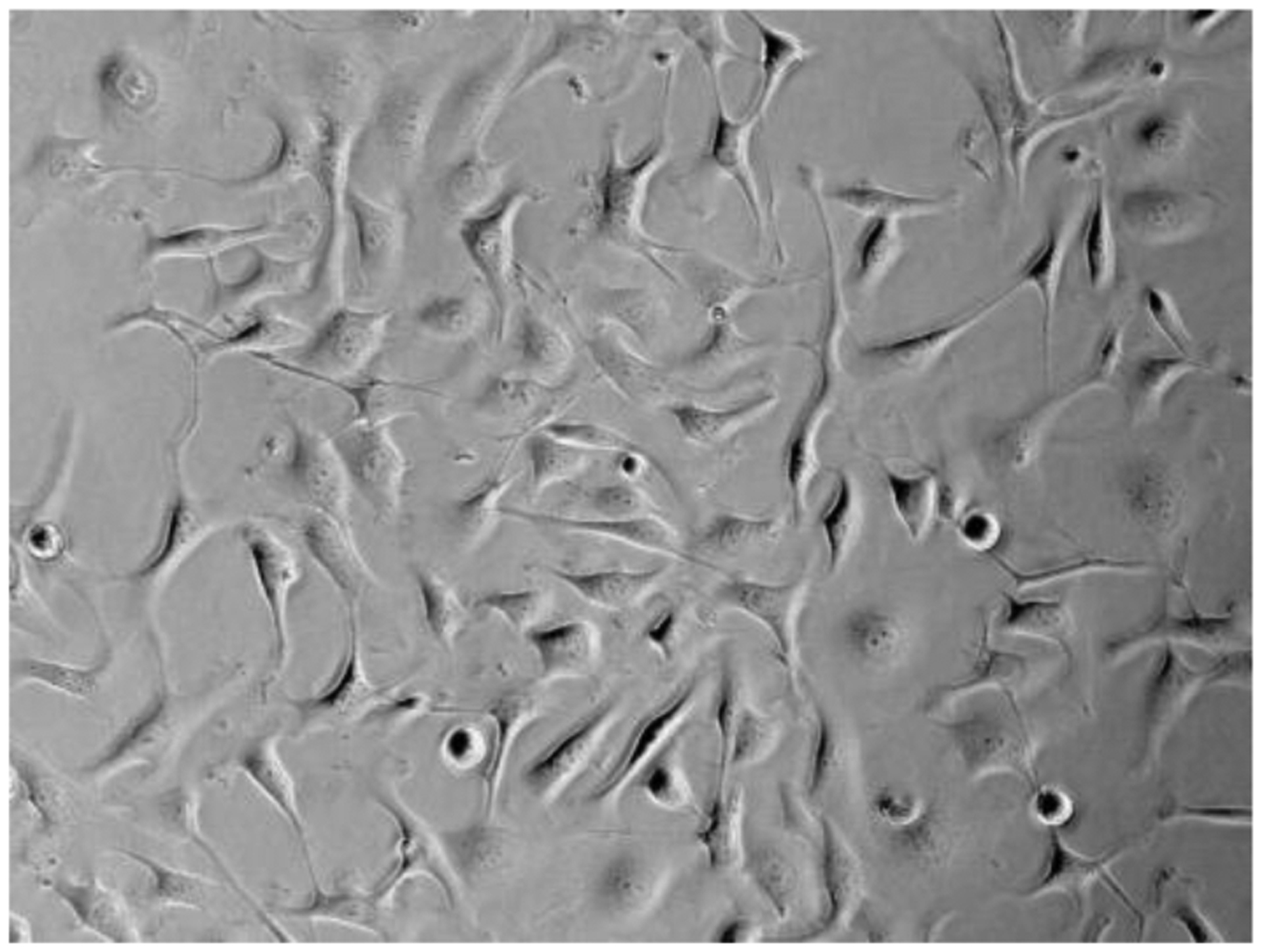
Optical image of SKOV3 cells 24 h post-plating on plastic petri dish.

To inquire the matrix effect on cell development, SKOV3 cells were cultured on these matrices. The morphology of the cells 24 h post-plating was examined by optical imaging (Figure 1d–f) and AFM imaging (Figure 1g–i). On a gelatin substrate, cells retained the squamous shape with no apparent alignment (Figure 1d,g). Scattered filopodia and lamellipodia were present around the periphery of a cell (Figure 1g), suggesting the tendency of cell migration in random directions. On a COL-CNT matrix, almost all cells elongated and formed bipolar spindle-like cellular extensions (Figure 1f). It is evident in Figure 1i that the cells polarized along the fibrils, and dense lamellipodia and filopodia were present at the two poles of the elongated cell body. Given the role of lamellipodia and filopodia in path finding and formation of focal adhesion complexes [32–34], it is expected that cell polarization and migration was facilitated in the fibril direction and was restrained in the perpendicular direction. On a collagen matrix, however, not all the cells were bipolar in shape (Figure 1e), and the cells shower more filopodia and lamellipodia extended from the cell periphery (Figure 1h), suggesting the potential cell migration in these directions. Note that the SKOV3 cells cultured on the plastic petri dish, which was also smooth and featureless but much harder than biopolymer matrices, demonstrated a similar cell morphology as those on gelatin (Figure A1).

**Figure 2.**
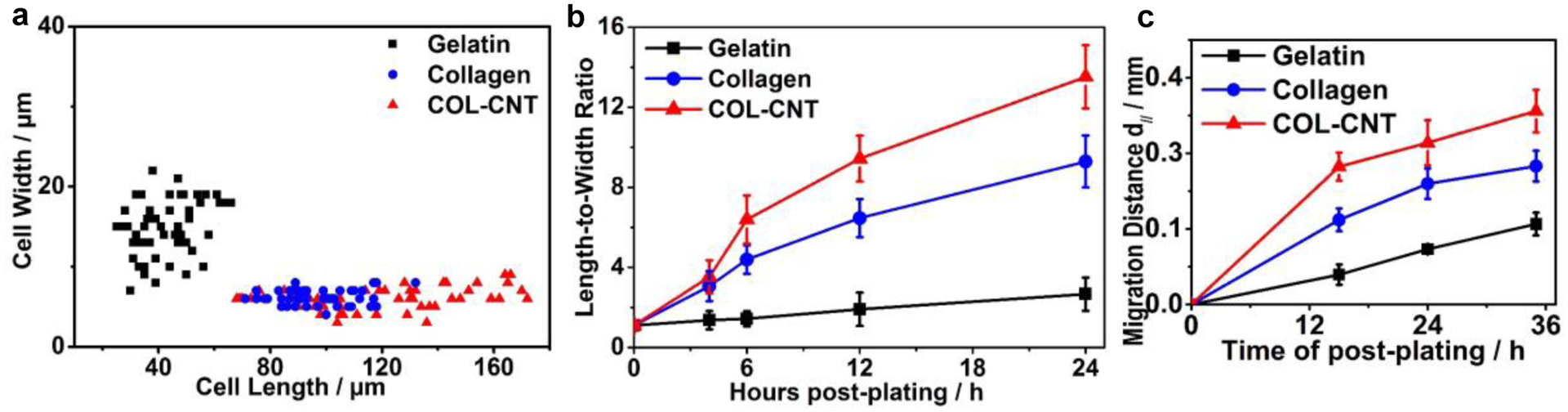
Examination of cell polarization and migration. (**a**) Scatter plot illustrating the correlation between the cell width and cell length of individual cells. The data were derived from more than 50 cells on each matrix type. (**b**) Change in the length-to-width ratio with time for cells cultured on the matrices. (**c**) Cell migration, characterized by the change in cell migration distance with time, on the matrices by the wound-healing assay.

The cell polarization on these matrices is compared quantitatively in the scatter plot in Figure 2a. On average, cells on COL-CNT and collagen fibrils were 4.0 times and 2.3 times longer than the cells on gelatin, respectively, and the cell width was reduced to half. The kinetics of cell polarization was characterized by the change in the cell length-to-width ratio over time (Figure 2b). Before seeding on a target matrix, cells were cultured to confluence on a plastic petri dish (at 0 h). The length-to-width ratio of these cells was 1.2 ± 0.2. Within 4 h post-plating on collagen and COL-CNT matrices, cells already began to polarize. However, the cells on gelatin were essentially unchanged in shape. With the increase in culture time, the cells polarized faster on COL-CNT than on collagen. After 24 h, the length-to-width ratio of cells on COL-CNT (13.5 ± 1.6) was 1.45 times higher than that on pure collagen (9.3 ± 1.3) and was 6.1 times higher than that on gelatin. The remarkably faster polarization of cells on COL-CNT suggests that the composite fibril matrix was favored over pure collagen to induce unidirectional cell growth.

**Figure A2.**
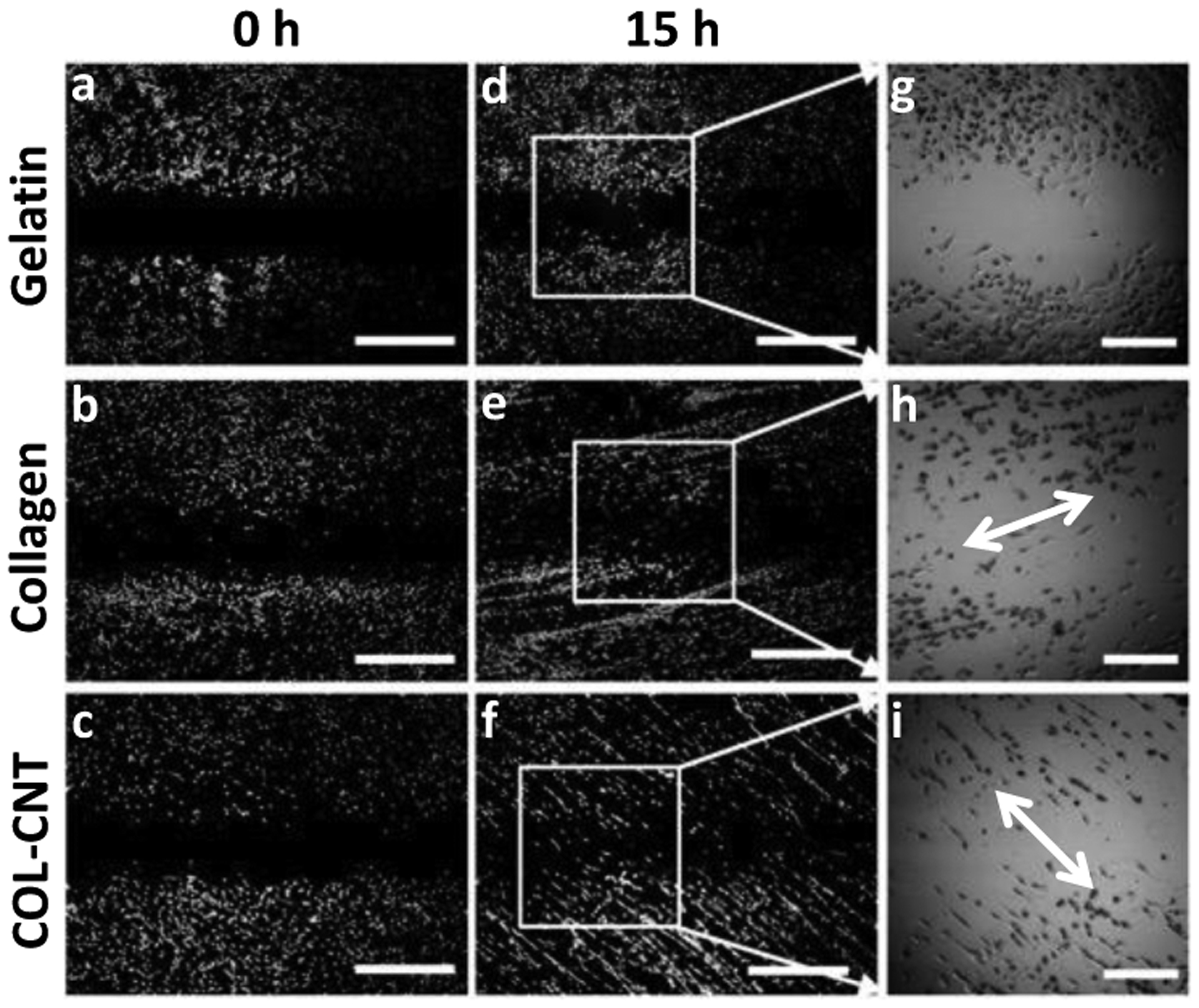
Characterization of cell migration on various matrices by the wound-healing assay. (**a**–**c**) Optical images of cells at the initial stage (0 h); (**d**–**f**) optical images of cells after 15 h migration; (**g**–**i**) enlarged images of (**d**–**f**) highlighting the cells at the gap region. Arrows indicate the direction of fibril alignment. Bar size of (**a**–**f**): 800 μm; bar size of (**g**–**i**): 300 μm. To investigate the matrix effect on cell migration, SKOV3 cells were seeded in two chambers separated by a defined gap of 500 μm. Time-lapse images were collected to monitor cell migration (Figure A2). Apparently, cells on collagen and COL-CNT protruded and migrated preferentially along the fibrils, while cells on gelatin migrated in random directions.

A wound-healing assay was employed to examine the matrix effect on cell migration (Figure 2c). Based on the time-lapse images to monitor cell diffusion and migration toward closing the gap, cells on collagen and COL-CNT migrated preferentially along the fibrils (Figure A2). The initial rates of cell migration (in the first 15 h) on gelatin, collagen, and COL-CNT were 3.5 ± 1.2, 10.0 ± 0.5, and 16.3 ± 0.9 μm/h, respectively. As migration continued, cells migrating from both sides of the gap merged at the center and accumulated. This hampered the cells’ further migration. Predictably, cells migrating faster appeared at the center earlier; consequently, they were impeded at an earlier time point. This is consistent with the observation that the hindrance occurred after 15 and 24 h for cells on COL-CNT and pure collagen, respectively. On gelatin, however, cells migrated at a constant, low rate even after 36 h. The result suggests that the aligned COL-CNT matrix accelerates cell migration.

### 3.2. Characterization of Fibril Stiffness and Cell Stiffness

**Figure 3.**
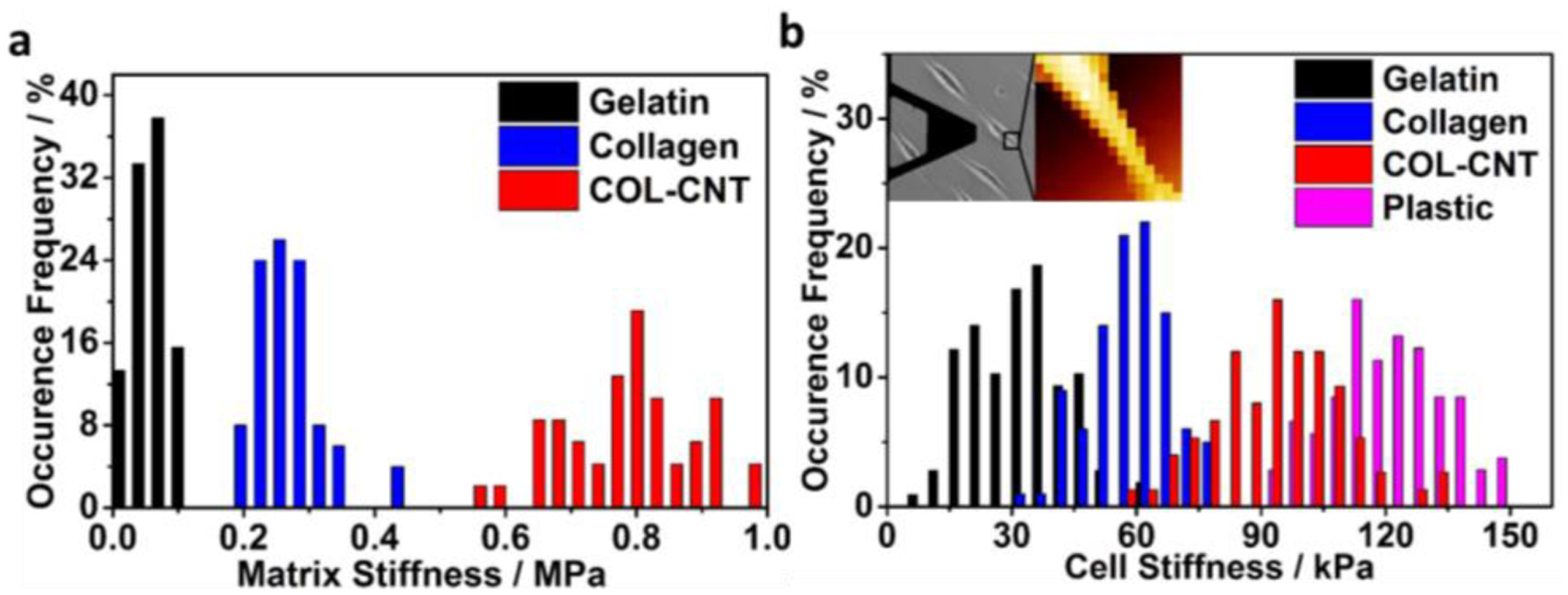
Statistical analyses of matrix stiffness and cell stiffness. (**a**) Histogram of E-value distribution for the matrices; (**b**) histogram of E-value distribution for cells cultured on the matrices for 3 days. The insets illustrate the phase-image-assisted elasticity measurement at the stress fiber-rich region of a targeted cell. Dimension of the phase image (left): 450 × 450 μm^2^; dimension of the force map (right): 35 × 35 μm^2^.

Due to the high tensile strength of CNT, the incorporation of CNT to collagen is expected to increase the matrix stiffness. The Young’s moduli (E-values) of gelatin, collagen, and COL-CNT fibrils were evaluated by the AFM nano-indentation method, and the data are summarized in the histogram in Figure 3a. The mean E-values for gelatin, collagen, and COL-CNT were 0.08, 0.27, and 0.84 MPa, respectively. The E-value of a plastic substrate was also measured (182.9 MPa) for reference. Evidently, COL-CNT fibrils were more rigid than the collagen fibrils and gelatin substrate, and the plastic substrate was strikingly stiffer compared to the protein and protein composite matrices.

It is conceivable that a stiff matrix exerts a high force on cells to activate stress fibers at a high level, leading to increased cell stiffness. When the stiffness of cells was examined, it was found that the mean E-values of cells on gelatin, collagen, and COL-CNT were 33, 59, and 94 kPa, respectively (Figure 3b). Noticeably, cells on plastic were even stiffer (119.7 kPa) than those on COL-CNT. However, these cells did not develop into the mesenchymal phenotype (see below).

### 3.3. Cell Adhesion

**Figure 4.**
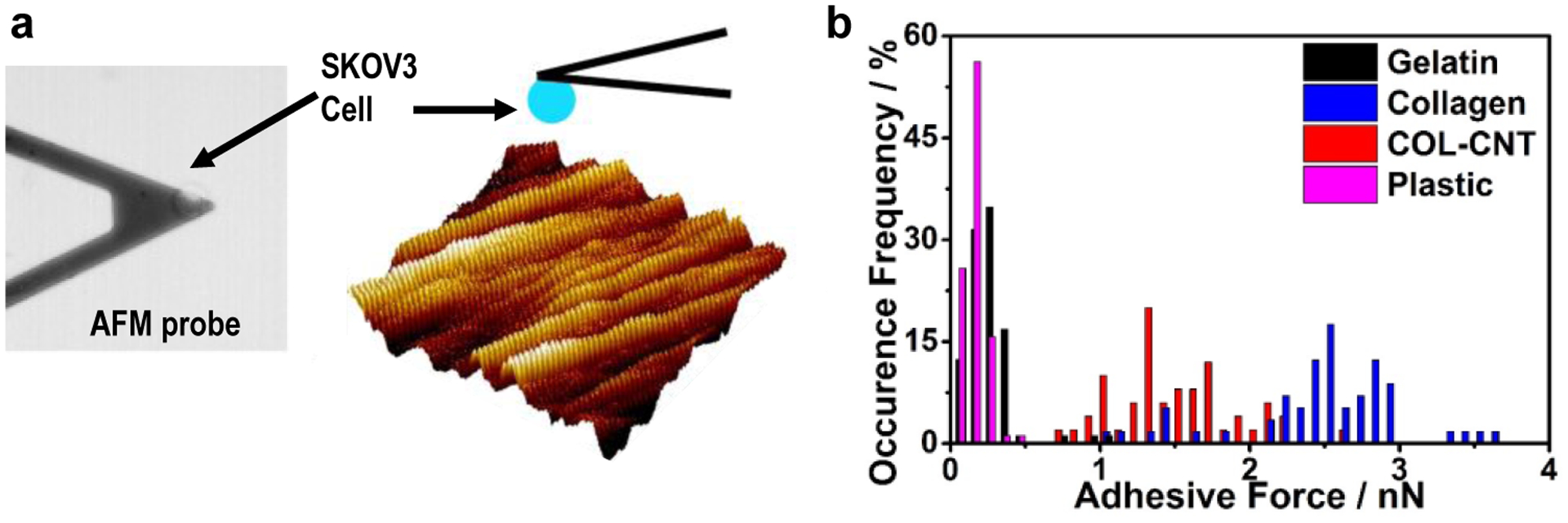
Cell adhesion on various matrices. (**a**) Illustration of the modification of a single cell on a PDL-coated AFM cantilever (phase image on the left) and the scheme (on the right) showing the cell adhesion measurement on a COL-CNT matrix. (**b**) Histogram of adhesion forces measured between individual cells and the selected substrates.

A matrix impinges on cell development through cell adhesion that is mediated by cell–matrix interaction. Cell adhesion was quantified by applying single-cell force spectroscopy, in which a SKOV3 cell was coated on an AFM probe to generate the force–distance curves on a target matrix (Figure 4a). Force curves measured on the matrices were statistically analyzed, and the results are illustrated in Figure 4b. The strength of cell adhesion on COL-CNT (1.5 ± 0.4 nN) was weaker than that on collagen (2.5 ± 0.5 nN), and the difference was significant (*p* < 0.00001). On the control substrates of gelatin and plastic, the strengths of cell adhesion were 0.23 ± 0.16 and 0.15 ± 0.06 nN, respectively, at least 6 times lower compared to that on collagen or COL-CNT.

**Figure 5.**
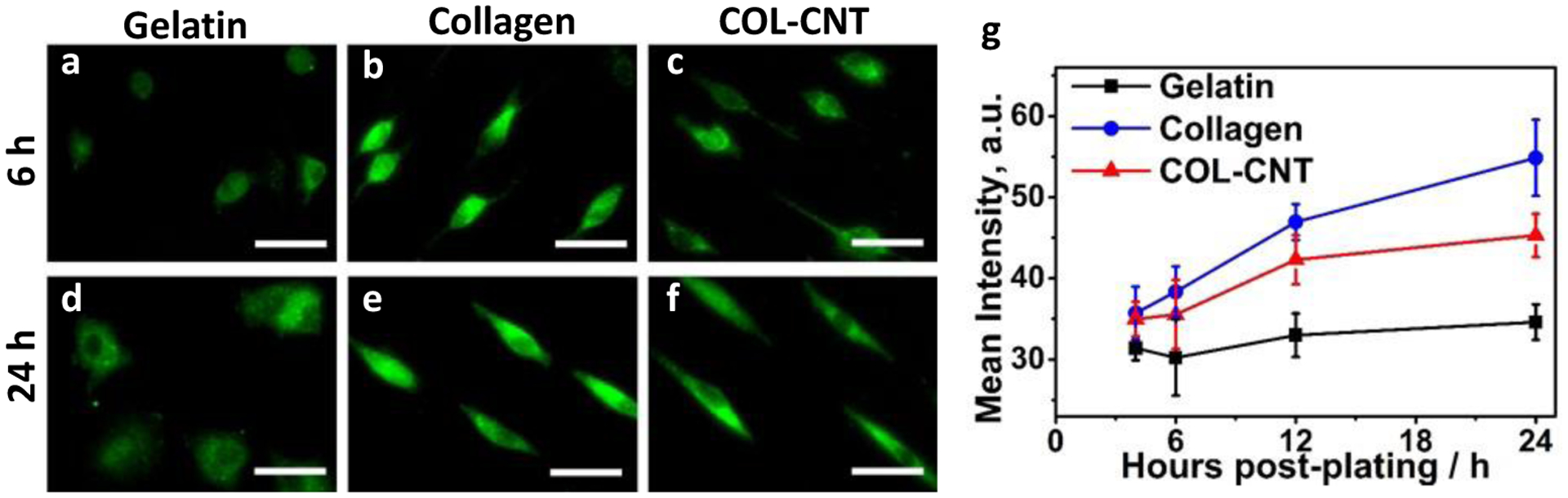
Time dependence of β−1 integrin expression in SKOV3 cells cultured on various matrices. (**a**–**f**) Immunofluorescent images of cells cultured on various matrices for 6 (**a**–**c**) and 24 h (**d**–**f**), respectively. Bar size: 115 μm. (**g**) ImageJ quantification of the change in β−1 integrin expression with time on the matrices.

It is known that cells bind to collagen through collagen–β1-integrin-specific interaction to form focal adhesion complexes that participate in the regulation of cell spreading, migration, growth, and survival [10,11,24,27,35]. Immunostaining of β−1 integrin was carried out to quantify its expression in SKOV3 cells at 4–24 h post-plating on the selected matrices (Figure 5). At any time-point, the expression of β−1 integrin in cells on COL-CNT was lower than that on pure collagen but was higher than that on gelatin. This is consistent with the strength of cell adhesion to the three matrices. It implies that the collagen–β1-integrin binding plays an essential role in the cell–matrix interaction. On the other hand, the β−1 integrin expression in cells on COL-CNT was shown to increase in the first 12 h of culture but changed marginally afterward (Figure 5g). This suggests that a stable interaction between the cells and the COL-CNT matrix was established early.

### 3.4. Collagen and COL-CNT Induced Cell Transformation

**Figure 6.**
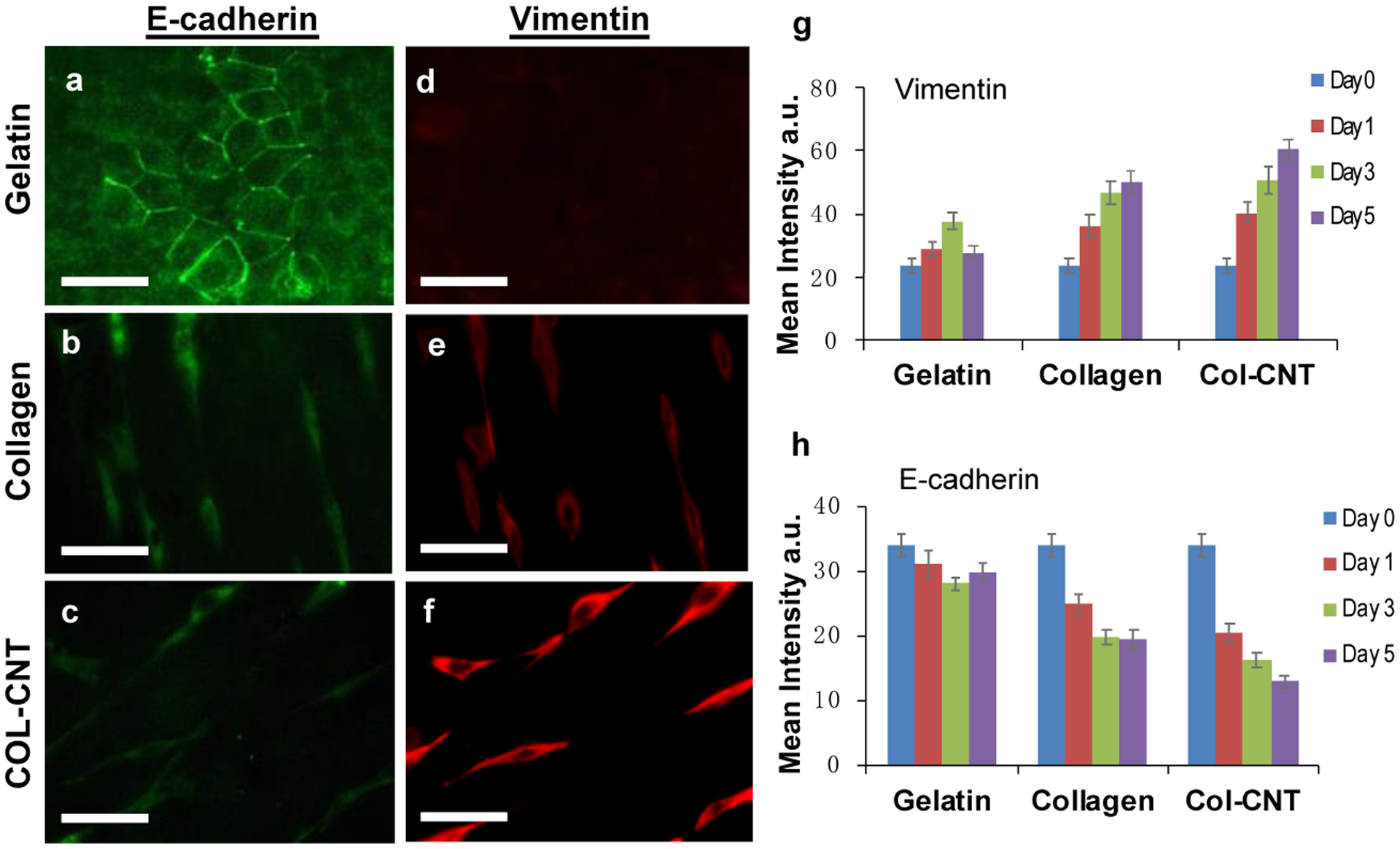
E-cadherin and vimentin expressions in SKOV3 cells cultured on the matrices. (**a**–**f**) Immunofluorescent images of SKOV3 cells staining against E-cadherin (**a**–**c**) and vimentin (**d**–**f**) on the matrices. Bar size: 193 μm. The cells were cultured on the matrices for 3 days. (**g**,**h**) ImageJ quantification of the expression levels of vimentin (**g**) and E-cadherin (**h**) in cells grown on the matrices at Day 0, 1, 3, and 5.

We speculated that on the aligned collagen and COL-CNT matrices, the epithelial-to-mesenchymal transition occurred when the squamous epithelial cells underwent a dramatic structural change to become bipolar, and they migrated significantly faster. A hallmark of EMT is the downregulation of epithelial biomarker E-cadherin and the upregulation of the mesenchymal biomarker vimentin [33,36]. Thus, the expression levels of E-cadherin and vimentin in SKOV3 cells on the matrices were monitored by immunofluorescence imaging (Figure 6a–f). Evidently, the cells on gelatin expressed E-cadherin at the highest level, whereas cells on COL-CNT expressed vimentin at the highest level. ImageJ analysis was carried out for images collected over 5 days to examine the change in the protein expression levels (Figure 6g,h). On both collagen and COL-CNT, the expression level of E-cadherin decreased with culture time, whereas the expression level of vimentin increased. The changes in COL-CNT were faster and more significant when compared to changes in collagen. On the contrary, the expression levels of both proteins in cells on gelatin showed marginal changes (*p* > 0.05). The results suggest that the EMT occurred in cells on both collagen and COL-CNT fibrils, and was more significant on COL-CNT.

**Figure 7.**
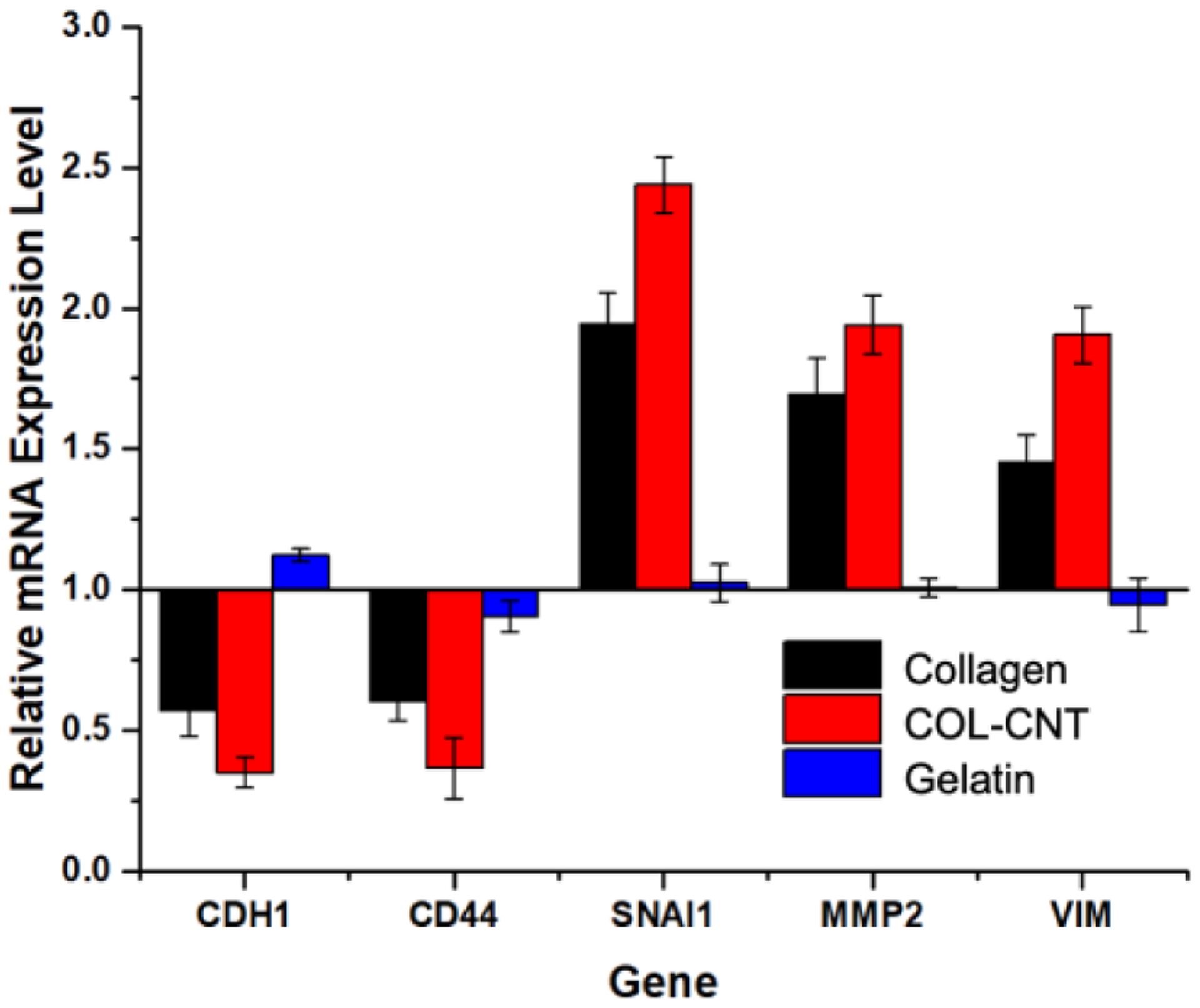
Gene expression profiles of SKOV3 cells grown on collagen, COL-CNT, and gelatin by Day 3. The expression of each gene in cells initially cultured on the plastic petri dish was used as a control to derive the relative mRNA expression.

RT-qPCR analysis of cells on collagen and COL-CNT was carried out to examine the changes at the gene level. The fold difference is relative to the gene expression of cells originally cultured on plastic petri dishes. CDH1 encodes E-cadherin and VIM encodes vimentin. Snail, a transcription factor that signals the EMT in many cancer types, is encoded by the SNAI1 gene. As shown in Figure 7, the downregulation of CDH1 and upregulation of VIM and SNAI1 were apparent in cells on both collagen and COL-CNT, with more remarkable changes in cells on COL-CNT. This consistently supports that COL-CNT prompted a more efficient EMT. We also monitored the expression levels of CD44 and MMP2. CD44 is a molecular predictor of survival in ovarian cancer. As a cancer stem cell marker, CD44 is closely relevant to tumor progression and metastasis [37–41]. It was reported that MMP2 expression was higher in benign tumors than in borderline and malignant tumors of ovarian cancer [42,43], and the cells were sensitive to chemotherapy [42]. We observed the downregulation of CD44 and upregulation of MMP2 in cells on both collagen and COL-CNT, and the changes were greater in cells on COL-CNT. On the other hand, in cells on the gelatin substrate, the expression levels of all genes barely changed. Thus, these cells retained the epithelial phenotype on the soft, featureless matrix.

To examine the susceptibility of the differentiated cells on collagen and COL-CNT, we treated the cells with the chemotherapy drug Gemcitabine, followed by viability tests. It was evident from the proliferation curves that the drug has the effect of suppressing cell proliferation. The viability of cells cultured on gelatin, collagen, and COL-CNT was found to be 58.2%, 72.4%, and 74.2%, respectively. While the cells on all substrates were responsive to the drug treatments, the cells on gelatin were more sensitive. However, the cells on collagen and COL-CNT demonstrated negligible differences in drug sensitivity.

## 4. Discussion

CNT has been extensively used as a component of biocomposite materials to create nanostructured scaffolds that provide unique features to modulate cell behaviors for tissue engineering and cancer treatment due to their chemical stability and exceptional mechanical strength [2,35,36]. In the current study, we generated the COL-CNT nanofibrils to mimic the collagen in the local environment of ovarian tumor stroma and revealed the unique matrix effect on modulating ovarian cancer cells.

While epitaxial growth of collagen fibrils on mica has been achieved and investigated, to the best of our knowledge, this is the first report of generating aligned COL-CNT composite nanofibrils by this approach. Compared with other methods to achieve aligned fibrillar collagen, the epitaxial growth method enables highly ordered, nearly identical, densely packed and nanometer-scale fibrils, resembling those in the ECM of soft tissues [14,15,44,45]. Consistent with our previous reports [9–11], CNT modified the structural framework of collagen at the molecular level. Compared with pure collagen fibrils, the COL-CNT nanofibrils were thicker and stiffer; additionally, COL-CNT presented a 2 nm longer D-period. This is ascribed to the peculiar alignment of CNT along the side of tropocollagen [11], which relaxes and flattens the helical coil of collagen fibrils, leading to the extension of the D-period. In collagen I, GFPGER (502–507) is the specific ligand sequence responsible for β−1 integrin binding. This sequence is present in the cell–matrix interaction domain in the overlap region of the collagen D-period [46]. A 2 nm increase in collagen D-period leads to a significant increase in the ligand spacing and, hence, a decrease in ligand density and a weaker cell adhesion. On the other hand, the alignment of CNT along the side of tropocollagen hinders the accessibility of the amino acids within the β−1 integrin binding region (i.e., GFPGER). This ligand presentation modulates integrin expression in cells and, subsequently, the formation of focal adhesion complexes, as well as the downstream integrin-mediated signaling cascades. This is consistent with the observation that lamellipodia or filopodia were rarely found along the sides of a cell on a COL-CNT matrix, whereas cells on a pure collagen substrate developed rich lamellipodia and filopodia on both sides of a cell. The extended lamellipodia or filopodia are known to sense cues and bind to collagen through integrin, developing stable focal contacts to assist cell adhesion and direct cell migration [32,33,47]. A lower adhesion of SKOV3 to COL-CNT is associated with a faster turnover of adhesion sites and, hence, a higher speed of spreading along the fibrils which favors the development of the spindle-shaped cell morphology and promotes faster migration. On the other hand, the addition of a miniscule amount of CNT resulted in a significant increase in collagen fibril stiffness owing to CNT’s exceptionally high tensile strength and toughness. A stiffer scaffold exerts a stronger force on the cells through the focal adhesions [48,49], inducing a greater level of cytoskeletal tension and, hence, a greater level of cell polarization and higher contractility.

The dramatic changes in cell shape and motility were accompanied by the downregulation of E-cadherin and the up-regulation of vimentin and SNAI1, hence the occurrence of an EMT [36–39]. Both protein level and gene level analyses support that the changes were more significant in cells on COL-CNT than in cells on pure collagen. Interestingly, the induced cells on the collagen and COL-CNT fibrils became less aggressive than the original cells and the cells cultured on gelatin, characterized by the reduced CD44 expression, increased MMP2 expression, and the lack of drug resistance. Noticeably, COL-CNT fibrils had a greater impact. We surmise that, while the cells underwent an EMT-like process on the collagen and COL-CNT fibrils, the fibrils induced drastic changes of the cells and likely caused transdifferentiation of the epithelial cancer cells to mature, less aggressive, and less potent cells along the path due to the extensive cell polarization in response to the unique matrix traits. The cells grown on COL-CNT were as susceptible to the chemotherapy drug as the cells grown on collagen fibrils. Thus, the presence of CNT in a collagen matrix, as the sole factor, is unlikely to cause an adverse effect on cancer patients if a controlled dose of CNT is used for drug delivery or tissue regeneration. Other factors in the tumor stroma, such as growth factors, signaling proteins, and cancer associated fibroblasts, may play concerted roles to influence the tumor malignancy and treatment outcome.

## 5. Conclusions

Taken together, epitaxial growth of the collagen-CNT composite was achieved to develop unidirectionally aligned, densely distributed, and unform nanofibrils. The ordered anisotropic matrix imposes unique biophysical and biochemical cues and offers a model system to evaluate the interplay of various matrix properties in modulating the cell–ECM interaction. Both the rigid, unidirectionally aligned nanofibrils and the unique presentation of β−1 integrin binding sites of COL-CNT promoted extensive cell polarization, fast migration, and decreased cell adhesion. They also transmitted the signal to promote the cell differentiation. The transformed cells were remarkably different from the original epithelial cells; they were less aggressive and lacked drug resistance. Information derived from the study could be potentially applied to develop new methods for modulating the stroma of carcinoma to effectively reduce the aggressiveness of the tumor cells.

## Data Availability

The data presented in this study are available on request from the corresponding author.
